# Distal Femoral Replacement for Fragility and Periprosthetic Fractures in a District General Hospital: A Five-Year Retrospective Cohort Study

**DOI:** 10.7759/cureus.97331

**Published:** 2025-11-20

**Authors:** Jack F Dufficy, James E Archer, Peter C Logan, Abdus S Burahee, Fahad Hossain, Thomas Moores

**Affiliations:** 1 Trauma and Orthopaedics, Walsall Manor Hospital, Walsall, GBR; 2 Trauma and Orthopaedics, Queen Elizabeth Hospital Birmingham, Birmingham, GBR

**Keywords:** distal femoral replacement, distal femur fracture, fragility fracture, periprosthetic femur fracture, surgical site infection

## Abstract

Background

Distal femur fractures in elderly or comorbid patients pose a significant challenge due to poor bone stock, complex fracture morphology, and limited tolerance for prolonged immobility. Distal femoral replacement (DFR) is increasingly utilised as an alternative to internal fixation, but evidence for its use outside tertiary centres remains limited.

Methods

We retrospectively reviewed all DFR procedures for trauma indications at a district general hospital (DGH) over a five-year period (2019-2024). Patient demographics, perioperative variables, infection rates, transfusion requirements, length of stay, and mortality were analysed.

Results

Twenty-four patients underwent DFR for non-oncological trauma, including native and periprosthetic distal femoral fractures. Surgical site infections (SSIs) occurred in 25% (n = 6) of cases, with 16.7% superficial and 8.3% deep infections. Median time to surgery was three days. A total of 83.3% of patients required blood transfusions. Median length of stay was 17.5 days. One-year mortality was 16.7%, and 90-day mortality was 8.3%. All discharged patients returned to their own homes.

Conclusion

DFR can be safely delivered in a DGH setting with infection and mortality rates comparable to published data from tertiary centres. Early surgery and full weight-bearing rehabilitation are achievable and may contribute to functional recovery in this vulnerable cohort.

## Introduction

Distal femur fractures, defined as injuries between the distal diaphyseal-metaphyseal junction and the articular surface of the femoral condyles, present a significant treatment challenge. These fractures are most commonly classified using the AO/OTA classification for native distal femur and the Su classification for periprosthetic fractures [1,2].

Recent epidemiological data show a 4.7-8.7 per 100,000 incidence of distal femur fractures with a bimodal peak incidence of younger patients who have sustained high-energy trauma and elderly patients sustaining low-energy fragility fractures [3]. The mean age at the time of fracture is 62 years, with an average male age of 44 and an average female age of 72 [3]. A significant majority of these fractures (97%) are from low-energy trauma in the geriatric population; most commonly (61%) due to a fall from standing height in female (66.6%) patients [3,4]. An epidemiological study found that the most common fracture morphology was AO/OTA type A (38.6%), and 28.7% of fractures were periprosthetic [4].

Surgical options include open reduction and internal fixation (ORIF) (e.g., locking plates, intramedullary nails) and distal femoral replacement (DFR) [3,5,6]. Non-operative treatment is limited to undisplaced fractures or patients unfit for anaesthesia [3,5]. Originally developed for orthopaedic oncology, DFR is now used for traumatic distal femur fractures and complex periprosthetic fractures [3,7,8]. The potential for immediate full weight bearing and rapid rehabilitation makes DFR an attractive option, particularly for frail or immobile patients. This expansion of indication has taken place in tertiary orthopaedic centres and large hospitals in which the expertise of use of a relatively high-risk and uncommon procedure is present [3,9,10].

Patients with distal femur fractures, especially in the geriatric population, are at significant morbidity and mortality risk as a result of highly invasive surgery in the context of limited physiological reserve, associated post-operative risk and loss of mobility. Mortality rates range from 13% to 35% at one year, depending on patient population and inclusion of oncology cases [11,12]. A systematic review found no significant difference in mortality and complication rates for geriatric patients treated with distal femur replacement or surgical fixation for traumatic distal femur fractures (oncology patients excluded) [13]. This review was limited by small cohorts in its constituent studies, which hindered the statistical significance of mortality and complication rate differences between DFR and ORIF groups. A 2022 cohort study comparing DFR with ORIF yielded increased revision-free survivorship of DFR patients at two-year follow-up, with 90% and 65% revision-free survivorship, respectively [14].

The inclusion of the DFR in the operative arsenal for geriatric distal femur fracture has been driven by the theoretical early mobilisation it can offer. Conversely, protected weight bearing is often necessary in early recovery for ORIF techniques to allow initiation of bone union. Better functional outcomes can be achieved, via the Parker mobility Index, at all post-operative stages up to one year post-discharge in over 65-year-olds [15]. In the geriatric population, this is especially pertinent to reduce their risk of hospital-acquired infection, loss of mobility, loss of independence and risk of post-operative mortality. In proximal femur fracture patients, who are similarly geriatric and clinically vulnerable, it is widely agreed that early full weight bearing is associated with better morbidity and mortality outcomes [16-19]. However, there is a lack of clear evidence when comparing DFR with ORIF for native and periprosthetic fractures in non-oncological, elderly patients, especially outside tertiary referral centres [9,20]. A systematic review of post-operative weight-bearing status in geriatric distal femur fracture fixation and its effect on mortality found no significant difference, and this may be due to data heterogeneity and poor study design [20].

In the last five years at our district general hospital (DGH) in the West Midlands (United Kingdom), we have surgically managed 66 non-oncological distal femur fractures locally, with 24 patients treated with DFR and 42 with ORIF.

Previously complex cases which were suitable for DFR have been referred to a local tertiary centre based on the existing referral pathway. This utilises a ‘hub and spoke’ model used in the National Health Service (NHS). However, access limitations and increasing demand have prompted the adoption of DFR in DGHs. Longer inpatient pre-operative waiting times worsen an already significant complication profile, with added risks of hospital-acquired infection and physiological deconditioning.

Our study aims to address this gap by reporting outcomes of DFR performed for trauma indications in a DGH setting. We evaluate post-operative morbidity, mortality, and complications, and assess the feasibility and safety of delivering this complex intervention outside of specialist centres.

## Materials and methods

Study design and setting 

This was a retrospective review of all patients undergoing DFR at a DGH in the West Midlands, United Kingdom. The study was registered with the local Clinical Audit Registration Management System (CARMS), and following institutional approval, the hospital’s patient databases were searched over a six-year period,  from January 2019 to December 2024. The hospital serves a population of 270,000 in the West Midlands [21] and traditionally refers complex cases to a nearby tertiary centre; however, since 2019, these complex distal femoral fractures are no longer referred and are instead managed locally. 

Inclusion and exclusion criteria

We included all adult patients undergoing DFR for trauma-related indications, including both native distal femur and periprosthetic fractures, who received an operation at our West Midlands DGH between 1st January 2019 and 31st December 2024. We excluded patients undergoing DFR for oncological or metastatic bone disease. A possible confounding variable identified was cases of distal femur fractures with trauma indications that were sent to a tertiary centre for surgery rather than managed locally, though none were identified in this period.

Surgical technique and perioperative management

We utilised a pre-operative bundle including two-step skin preparation with alcoholic chlorhexidine, routine administration of intravenous antibiotics (teicoplanin and gentamicin as per our trust guidelines), patient warming, administration of intravenous tranexamic acid and application of a tourniquet. An extensile medial parapatellar approach was utilised in all cases. A fully cemented endoprosthesis was used in all cases. All wounds were closed with skin clips. Prophylaxis of venous thromboembolism was provided in line with our trust guidance with compression stockings on the contralateral leg and weight-based prophylactic dosing of subcutaneous enoxaparin for 14 days. Any patient on pre-existing anticoagulation was converted back to their original anticoagulants after clinical review deemed it suitable.

Data collection  

Data were obtained from theatre logs, patient records, and follow-up documentation. Variables collected included patient age, sex, time from admission to surgery, transfusion requirements, length of stay, discharge destination, readmission rates, surgical site infection (SSI), and mortality at 30 days and one year. At the time of writing, all patients were contacted for a virtual follow-up. 

Outcomes  

The primary outcome was SSI. This was further classified into superficial and deep infection, with superficial infection defined as requiring antibiotic treatment only and deep infection requiring surgical management [22]. Secondary outcomes included time to surgery, transfusion requirement, hospital stay, discharge location, and mortality. 

Statistical analysis  

Due to the limited sample size, only descriptive statistics were used. Means, medians, and ranges were calculated where appropriate.

## Results

Between 2019 and 2024, 24 patients underwent DFR for non-oncological indications. The median patient age was 80 years (range: 52-91), and the majority were female (87.5%). Demographic data is summarised in Table 1.

**Table 1:** Patient demographics and outcome data.

Age (years)	Sex (M/F)	Admission date	Operation date	Time to surgery	Admission Hb	Postop Hb	Hb drop	Transfusion requirement	Length of stay	Discharge location (0 = own home, 1 = step down)	30-day readmission	Infection (0 - none, 1 - abx only, 2 - surgical Mx)	Reoperation notes	Follow-up (months)	Mortality	Mortality interval
91	M	2/28/2021	3/1/2021	1	115	81	34	2 units postoperatively	18	0	1	0	-	20	5/26/2023	816
85	M	11/27/2021	11/29/2021	2	137	110	27	N/A	19	0	0	0	-	2	3/13/2023	469
89	F	10/18/2021	10/20/2021	2	78	90	-12	2 units preoperatively, 3 units postoperatively	8	0	0	2	2 stage revision 01/22	3	N/A	N/A
81	F	11/26/2019	11/29/2019	3	90	104	-14	4 units preoperatively	17	0	0	0	-	3	5/31/2025	2010
83	F	12/21/2019	12/30/2019	9	132	100	32	N/A	24	0	0	1	-	5	N/A	N/A
60	F	5/24/2020	5/27/2020	3	142	100	42	N/A	8	0	0	0	-	9	N/A	N/A
87	F	5/30/2021	6/1/2021	2	110	80	30	2 units postoperatively	18	0	1	2	DAIR 09/07/21	35	5/13/2024	1077
67	F	8/11/2021	8/12/2021	1	121	78	43	2 units postoperatively	14	0	0	0	-	32	N/A	N/A
78	F	5/31/2020	6/5/2020	5	125	91	34	2 units postoperatively	12	0	0	0	-	1	N/A	N/A
77	F	12/12/2020	12/18/2020	6	82	109	-27	2 units preoperatively, 2 units postoperatively	35	N/A	N/A	0	-	1	1/20/2021	33
84	F	5/5/2021	5/7/2021	2	131	91	40	2 units postoperatively	13	N/A	N/A	0	-	0	5/18/2021	11
72	F	3/17/2022	3/18/2022	1	138	105	33	N/A	12	0	0	1	-	32	N/A	N/A
88	F	6/21/2022	6/22/2022	1	98	67	31	2 units postoperatively	14	0	0	0	-	11	N/A	N/A
87	F	7/4/2022	7/5/2022	1	131	104	27	1 unit postoperatively	43	0	0	0	-	14	N/A	N/A
87	F	7/2/2022	7/6/2022	4	80	91	-11	2 units preoperatively, 4 units postoperatively	94	0	0	1	-	7	2/2/2023	211
70	F	4/7/2023	4/12/2023	5	118	96	22	1 unit preoperatively, 1 unit postoperatively	23	0	0	0	-	4	N/A	N/A
86	F	5/29/2023	6/1/2023	3	121	76	45	1 unit postoperatively	14	0	0	0	-	0	N/A	N/A
84	F	1/16/2024	1/26/2024	10	95	81	14	2 units preoperatively, 1 unit postoperatively	28	0	0	0	-	6	N/A	N/A
82	F	4/14/2024	4/19/2024	5	124	102	22	1 unit postoperatively	32	0	0	0	-	5	9/4/2024	138
52	M	6/10/2024	6/17/2024	7	129	76	53	1 unit preoperatively, 2 units postoperatively	19	0	0	0	-	0	N/A	N/A
78	F	6/21/2024	6/24/2024	3	102	97	5	4 units preoperatively	14	0	0	0	-	3	N/A	N/A
69	F	10/17/2024	10/23/2024	6	91	82	9	3 units preoperatively	15	0	0	0	-	5	N/A	N/A
79	F	10/19/2024	10/25/2024	6	135	83	52	2 units postoperatively	26	0	0	1	-	5	N/A	N/A
78	F	1/12/2022	1/14/2022	2	129	73	56	2 units postoperatively	17	0	0	0	0	36	N/A	N/A

Primary outcome

*Infection * 

Six patients (25%) developed a postoperative SSI. Four infections (16.7%) were superficial and treated successfully with antibiotics alone. Two cases (8.3%) required return to theatre: one for debridement, antibiotics, and implant retention (DAIR), and one for two-stage revision at the local tertiary centre. 

Secondary outcome

*Surgical Timing * 

Time from admission to definitive surgery ranged from one to 10 days. The median was three days, and the mean was four days.

*Transfusion Requirements * 

Twenty of 24 patients (83.3%) required transfusion of packed red cells. All patients with admission haemoglobin <100 g/L were transfused preoperatively. Some patients with normal haemoglobin were also transfused based on anticipated blood loss. Of those who did not receive a preoperative transfusion of packed red cells, postoperative Hb drop ranged from 22 to 52 g/L, with an average loss of 35 g/L. 

Length of Stay and Discharge

Inpatient stay ranged from eight to 94 days (median: 17.5; mean: 22). All patients who survived to discharge returned to their own homes. 

Mortality

There were two inpatient deaths (8.3%), at 11 and 32 days postoperatively, respectively. Causes of death were pulmonary emboli, complicated by COVID pneumonia, and a spontaneous upper gastrointestinal bleed. Ninety-day mortality was 8.3% (two patients), and one-year mortality was 16.7% (four patients).

## Discussion

DFR is an increasingly recognised treatment option for complex distal femur fractures, particularly in elderly and comorbid patients for whom internal fixation may not offer optimal outcomes. The present study evaluated outcomes from a DGH, providing real-world evidence on the safety and feasibility of DFR in a non-tertiary setting. Our findings demonstrate postoperative infection and mortality rates comparable to those reported in the literature from high-volume specialist centres, with encouraging discharge outcomes.

Our median and mean time to discharge are 17.5 days and 22 days, respectively. All patients were discharged back to their own homes, and we do not have a dedicated rehabilitation ward or facility within our hospital. Therefore, our length of stay is longer than we might hope for. However, with all patients who were discharged returning to their own home, this demonstrates that further inpatient rehabilitation was not required after this length of stay. All patients received daily review from the therapies and medical teams to ensure they were progressing, and planning for their discharge could be arranged. The mean length of stay of our neck of femur fracture cohort at the same hospital between January 2019 and December 2024 is 19.5 days. This is a comparable cohort who have been through a shorter procedure with quicker recovery to mobilisation. We are therefore reassured that our length of stay for patients undergoing DFR is not excessively high.

Geriatric distal femur fractures are associated with significant morbidity and mortality, not dissimilar to hip fractures. The literature consistently highlights one-year mortality rates ranging from 13% to 35% following surgical treatment, depending on comorbidity burden, delay to surgery, and postoperative complications [5,6]. In our cohort, the 90-day and one-year mortality rates were 8.3% and 16.7%, respectively, aligning with the findings of Garabano et al., who reported a one-year mortality of 20% in a similar cohort treated with DFR or internal fixation [15]. 

The historical standard of care for distal femoral fractures has been internal fixation using locked plates or retrograde intramedullary nails. However, poor bone quality, fracture comminution, and the requirement for protected weight-bearing often result in delayed union, implant failure, and prolonged rehabilitation in elderly patients [23]. DFR provides immediate structural stability, allowing full weight-bearing postoperatively, which is critical in mitigating the risk of complications related to immobility such as pneumonia, pressure sores, and venous thromboembolism [3,6,8,15,20]. This theoretical advantage is supported by a systematic review, which reported improved early functional outcomes in patients undergoing DFR compared to fixation [13]. Although increasing in popularity in the context of trauma, DFR is known to be associated with a significant complication rate, quoted as up to 36% as quoted by some existing literature [11,12]. The German Arthroplasty Registry (EPRD) study by Lützner et al. found that patients undergoing DFR for periprosthetic fractures had a 16.8% revision rate at one year and a 25% mortality rate [10]. This underscores the importance of careful patient selection, meticulous surgical technique, and appropriate follow-up. In our series, we did not encounter any early mechanical failures or aseptic loosening, though long-term follow-up beyond one year will be necessary to assess implant survivorship and late complications. 

Infection remains a significant concern with DFR, with deep prosthetic joint infection rates cited as high as 13% in some series [24]. Our overall SSI rate was 25%, but only two cases (8.3%) were deep infections requiring surgical intervention, comparable to the 10-12% deep infection rates reported in the literature [9,24]. Importantly, the presence of experienced arthroplasty surgeons, appropriate perioperative protocols, and access to microbiological and infectious diseases support services are likely key in managing and mitigating infection risk. 

As with all such cases, open discussions with patients and their families about the risks of such surgery are essential. This current piece of work, along with others, can help to equip surgeons with accurate information to help patients and their families make informed decisions, while weighing the risks of complications and the risk of mortality. 

The viability of performing DFR in DGHs has been questioned due to concerns regarding case complexity, complication management, and resource limitations. However, our study supports the notion that with appropriate surgical expertise, implant availability, and perioperative infrastructure, DFR can be safely delivered outside tertiary centres. Our median time to surgery was three days, which compares favourably to the often-prolonged preoperative wait associated with inter-hospital transfers. Avoiding these delays is critical, as time to surgery over 72 hours was associated with significantly increased mortality in distal femur fracture patients [12]. 

Blood loss is a recognised intraoperative challenge during DFR, and transfusion requirements are common. In our series, over 80% of patients received a transfusion, highlighting the need for careful perioperative planning and optimisation. While transfusions carry inherent risks (including transfusion reactions, immunosuppression, and infection), optimising haemoglobin is important for wound healing and mobilisation. A recent systematic review suggested that perioperative anaemia is independently associated with worse orthopaedic outcomes, including delayed wound healing and longer hospital stay [25]. Preoperative transfusion protocols, especially for patients presenting with anaemia, should be standardised in institutions performing DFR. From our small cohort, we were unable to ascertain if the use of transfusions had any effect on the postoperative infection risks, but we did not see any complications related to transfusion use. 

A key benefit of DFR is the facilitation of early mobilisation. While we did not formally assess functional outcomes using validated tools such as the Parker Mobility Score [15], all patients discharged from our unit returned to their own homes, suggesting a satisfactory return to baseline function or independence. Other studies assessing patients undergoing DFR had better Parker Mobility Scores at discharge and at one-year follow-up compared to those who underwent ORIF, further supporting its utility in promoting functional recovery [13]. Given that early weight bearing is associated with improved outcomes in hip fracture populations [16], it is reasonable to extrapolate similar benefits in distal femur fractures, although more direct evidence is still needed. 

There is also a notable absence of consensus guidelines on the indications for DFR in the setting of trauma. The current literature is dominated by retrospective studies and registry data, with few prospective or randomised trials directly comparing DFR with ORIF. As a result, treatment decisions are often guided by surgeon preference, institutional expertise, and patient-specific factors rather than standardised algorithms. Some proposed indications for DFR include failed previous fixation, fracture non-union, severe osteopenia, comminuted intra-articular fractures, and patients unable to comply with non-weight-bearing restrictions [6,8]. Prospective multicentre studies are urgently needed to better define the role of DFR in these contexts. 

Our study is limited by its retrospective design, small sample size, and lack of a control group. Furthermore, functional outcomes were inferred from discharge destination rather than directly measured using validated instruments. Selection bias is also possible, as patients selected for local DFR may have been deemed more suitable or lower risk than those referred to tertiary centres. Whilst we have attempted to contact all patients at the time of writing, this remained impossible in the case of two patients, and therefore, their long-term outcome cannot be confirmed. Nonetheless, our results reflect real-world practice in a resource-limited environment and offer valuable insights into the expanding role of DFR in non-specialist settings. 

## Conclusions

Our findings add to the growing body of evidence supporting DFR as a viable option for managing complex distal femur fractures in elderly patients. Infection and mortality rates were within acceptable limits, and the ability to provide early, definitive surgical intervention within a DGH setting helped circumvent the delays often associated with tertiary referral. These data advocate for the decentralisation of complex arthroplasty care, provided that adequate training, resources, and support systems are in place.
